# Performance of Polymerase Chain Reaction Analysis of the Amniotic Fluid of Pregnant Women for Diagnosis of Congenital Toxoplasmosis: A Systematic Review and Meta-Analysis

**DOI:** 10.1371/journal.pone.0149938

**Published:** 2016-04-07

**Authors:** Christianne Terra de Oliveira Azevedo, Pedro Emmanuel A. A. do Brasil, Letícia Guida, Maria Elizabeth Lopes Moreira

**Affiliations:** 1 Instituto Nacional de Saúde da Mulher, da Criança e do Adolescente Fernandes Figueira (IFF/Fiocruz); 2 Instituto Nacional de Infectologia Evandro Chagas (Ipec/Fiocruz); Hospital Authority, CHINA

## Abstract

**Introduction:**

Congenital infection caused by *Toxoplasma gondii* can cause serious damage that can be diagnosed *in utero* or at birth, although most infants are asymptomatic at birth. Prenatal diagnosis of congenital toxoplasmosis considerably improves the prognosis and outcome for infected infants. For this reason, an assay for the quick, sensitive, and safe diagnosis of fetal toxoplasmosis is desirable.

**Goal:**

To systematically review the performance of polymerase chain reaction (PCR) analysis of the amniotic fluid of pregnant women with recent serological toxoplasmosis diagnoses for the diagnosis of fetal toxoplasmosis.

**Method:**

A systematic literature review was conducted via a search of electronic databases; the literature included primary studies of the diagnostic accuracy of PCR analysis of amniotic fluid from pregnant women who seroconverted during pregnancy. The PCR test was compared to a gold standard for diagnosis.

**Results:**

A total of 1.269 summaries were obtained from the electronic database and reviewed, and 20 studies, comprising 4.171 samples, met the established inclusion criteria and were included in the review. The following results were obtained: studies about PCR assays for fetal toxoplasmosis are generally susceptible to bias; reports of the tests’ use lack critical information; the protocols varied among studies; the heterogeneity among studies was concentrated in the tests’ sensitivity; there was evidence that the sensitivity of the tests increases with time, as represented by the trimester; and there was more heterogeneity among studies in which there was more time between maternal diagnosis and fetal testing. The sensitivity of the method, if performed up to five weeks after maternal diagnosis, was 87% and specificity was 99%.

**Conclusion:**

The global sensitivity heterogeneity of the PCR test in this review was 66.5% (I^2^). The tests show low evidence of heterogeneity with a sensitivity of 87% and specificity of 99% when performed up to five weeks after maternal diagnosis. The test has a known performance and could be recommended for use up to five weeks after maternal diagnosis, when there is suspicion of fetal toxoplasmosis.

## Introduction

Toxoplasmosis is a disease that is endemic globally and is caused by *Toxoplasma gondii*, an obligate intracellular protozoan parasite. Its prevalence varies widely from place to place, especially when related to health conditions and socio-economic indices [1,2]. In some regions of Brazil the prevalence of toxoplasmosis in women of reproductive age reaches 65.8% [3].

*T*. *gondii* infection is acquired primarily through ingestion of cysts in infected, undercooked meat or oocysts that may contaminate soil, water, and food [2]. Infection by *T*. *gondii* is typically mild or subclinical in healthy humans. However, infection during pregnancy puts the fetus at risk of congenital infection, which can cause serious lesions that can be diagnosed in utero or at birth [4]. The risk of transmission increases during pregnancy, but the severity of the disease in the fetus decreases.

Classical signs, such as hydrocephalus, chorioretinitis, and intracranial calcifications (Sabin’s triad), may not be present at birth; however, sequelae resulting from undiagnosed disease appear in adolescence or adult life. Undiagnosed and untreated patients develop irreversible lesions, especially in the eyes and brain [5].

Prevalence of congenital infection ranges from 0.1 to 0.3 per 1000 live births. The maternal–fetal transmission rate increases with gestational age at maternal seroconversion, from less than 15% at 13 weeks of gestation to over 70% at 36 weeks[6]. Although it is not clear whether there are advantages to treating congenital infections, some evidence suggests that treatment can reduce the risk of serious clinical injuries and neurological and ocular damage [4].

The prenatal diagnosis of congenital toxoplasmosis has considerably improved the prognosis and outcome for infected children [7]. Maternal serologic screening for prenatal toxoplasmosis detection is an important tool that allows for the adoption of prophylactic and therapeutic measures, reducing the rate of vertical transmission and damage to the fetus’ development [8]. The control programs for congenital toxoplasmosis vary throughout the world, and there is no consensus regarding the benefit of universal screening of toxoplasmosis during pregnancy.

In some European countries, such as France, monthly serologic screening of susceptible pregnant women has been recommended since 1992. If a serologic exam indicates acute infection, maternal treatment with spiramycin is initiated in an attempt to prevent transmission to the fetus. If fetal infection is confirmed by polymerase chain reaction (PCR) analysis of the amniotic fluid, spiramycin is replaced by a combination of pyrimethamine, sulfadiazine, and folinic acid [9].

A recent study [10] evaluated the impact of monthly serologic screening on the reduction of the transmission rate and improvement of the clinical results at age three and found that the sequelae of toxoplasmosis in children of women in whom the infection is identified and treated early during pregnancy are seldom severe, highlighting the benefits of prenatal screening.

In Brazil, prenatal screening is suggested but is a non-mandatory public policy with protocols that vary by region, thereby lacking uniformity [8]. Fetal testing is indicated when maternal seroconversion during pregnancy is confirmed and can be accomplished using direct tests, such as amplification of nucleic acid sequences in amniotic fluid by PCR or parasite isolation from amniotic fluid [11].

The interpretation of diagnostics for congenital toxoplasmosis using serological tests is not a trivial task due to the kinetics of emergence and the fact that transplacental transmission is highly variable [12,13]. The inoculation of amniotic fluid in mice, while having higher sensitivity and specificity, requires three to six weeks, and the animals must be kept in facilities [14].

Perhaps the greatest advance in the prenatal diagnosis of fetal infection with *T*. *gondii* has been the use of PCR analysis of amniotic fluid instead of umbilical cord blood samples [2]. The detection of the parasite's DNA does not depend on the patient’s immunological status, and this assay can be applied to a wide variety of samples and allows for comparatively rapid diagnostic results. The first qualitative PCR protocol for *Toxoplasma* was published in 1989 by Burg et al. [15]. He described the amplification of a sequence belonging to the gene B1, which is repeated 35 times in the parasite’s genome. Since then, many teams have developed PCR protocols for the detection of different target sequences of the parasite’s DNA. However, the proliferation of qualitative PCR protocols has resulted in considerable heterogeneity in the tests’ execution. The advent of quantitative PCR in the last decade promotes a smaller diversity among protocols. In addition, it allows the parasite load in a sample to be quantified [5,16].

Against this background (i.e., the greater technical variability in the execution of PCR and the possibility of method standardization using quantitative PCR), the goal of this investigation was to systematically review the literature on PCR performance when using amniotic fluid for the antenatal diagnosis of congenital toxoplasmosis.

## Materials and Methods

### Criteria for Study Inclusion

Studies on the use of PCR for fetal toxoplasmosis diagnosis were identified according to the following criteria: (a) original studies that included pregnant women with evidence of acute infection by *T*. *gondii* during pregnancy, (b) studies that evaluated the performance of PCR analysis of amniotic fluid, cord blood, or a pregnant woman’s blood, (c) studies that compared the PCR results with any “gold standard” or test accepted as better evidence, and (d) studies that reported the sensitivity and specificity or presented data to calculate these parameters. Summaries in any language were accepted for the initial classification. For full texts, the inclusion was restricted to the following languages: Portuguese, French, English, Spanish, and Italian. There was no limit on the paper’s disclosure date for inclusion.

### Criteria for Study Exclusion

The following exclusion criteria were applied: (a) studies that considered other populations (i.e., not pregnant women) or ones in which testing for toxoplasmosis was not related to fetal disease, (b) reviews, (c) editorials, (d) consensuses, protocols, and guides for clinical practice, (e) case reports, (f) congress summaries, (g) validation of lab methods, (h) studies in which the PCR data were used only to assess the treatment outcome and (i) neonatal or postnatal diagnostic investigations.

### Sources of Information

The initial search was conducted using the following databases: PUBMED, EMBASE, SCOPUS, WEB OF SCIENCE, and LILACS. The bibliographies of articles selected for full-text reading and of review articles were also checked to identify relevant works that had not been found in the electronic search. Our first search in the database was on January 25, 2012, and the last search on August 12, 2015. Initially, a search in MEDLINE with different combinations of terms, either in the title or in the summary and keywords, was conducted to identify terms capable of retrieving articles about the theme of interest that were already in the investigator’s possession. Details on the search strategy adopted to identify original studies are reported in S1 Text.

### Selection of Studies

After the application of search strategies for remote databases, the identified references were stored in bibliographical data management program for exclusion of replicas and to organize the summaries. The summaries were read independently by two reviewers, who met to resolve disagreements, selected the texts to be read in full, and extracted data. This last step was performed by three reviewers independently; one reviewer read all of the texts, whereas the other two read only parts of them. A second meeting was held to resolve disagreements regarding the inclusion and exclusion criteria, and the texts to include in the review were selected.

### Verification of Data

A form for data capture was designed and tested specifically to be used during the reading of full texts, reported in S2 Text. The test of the form consisted of each reviewer extracting data from different texts with at least two rounds of discussion about the items contained in the form and about the workflow. The form included the question contained in QUADAS 2 (Quality Assessment of Diagnostic Accuracy Studies) to evaluate the risk of bias. The form was composed of 66 questions divided into five sections: text identification, full text eligibility, risk of bias evaluation by QUADAS 2, study or sample characteristics, and test data.

### Assessed Items

The following were evaluated for each study: pregnancy age, nationality of the subjects, pregnancy stage at acute toxoplasmosis diagnosis, time between diagnosis of maternal infection and testing for fetal toxoplasmosis, proportion of pregnant women who received treatment for toxoplasmosis before fetal testing, time between the beginning of treatment and fetal testing, and whether there was evidence that the pregnant woman’s previous treatment influenced the result of the index test.

The index test was defined as the PCR analysis of amniotic fluid of pregnant women acutely infected by T. gondii during pregnancy. Acute maternal infection was defined as change from negative to positive specific immunoglobulin G (IgG) antibodies or a significant increase in IgG with concomitant high immunoglobulin M (IgM) titers [10].

Clinical follow-up of the child during its first year was considered the standard reference test. Diagnosis of congenital toxoplasmosis was confirmed when the specific antibodies (i.e., IgM, IgA or IgG) were detected in the newborn’s blood and persistence after the first year of life of specific IgG. Congenital toxoplasmosis absence was defined by negative IgG results in the absence of treatment at 12 months of age [10].

### Risk of Bias

Evaluation of the risk of bias in the full texts included in the review was conducted independently by the reviewers during data extraction using the QUADAS 2 [17]. This tool is recommended for use in systematic reviews of diagnostic performance by the Agency for Healthcare Research and Quality, Cochrane Collaboration, and the U.K. National Institute for Health and Clinical Excellence. It covers four domains: selection of patients, index tests, standard references, and time and flow (i.e., patients’ flow through the study and the timing of the index test and standard reference). Each domain is evaluated in terms of the risk of bias; the first three domains are also evaluated for applicability. This tool allows for a more transparent classification of the risk of bias and the applicability of the studies to assess diagnostic precision.

### Summary Measurements and Synthesis of the Results

The data were analyzed using the R-project statistical software, available at http://www.r-project.org/ [18] with the mada and meta packages. The statistical analysis consisted of calculations of estimates for sensitivity, specificity, and diagnostic odds ratio (DOR), with 95% confidence intervals. Combined summary measurements were calculated with fixed effect models for sensitivity and specificity and with random effects models using the DerSimonian-Laird method for sensitivity, specificity and DOR. Summary sensitivity and specificity measurements combined in the bivariate model proposed by Reitsma were also estimated with the respective confidence intervals. A summary ROC curve (SROC) and its area under the curve (AUC) were estimated by the method proposed by Rutter and Gatsonis. All of the analyses were conducted for data corresponding to the diagnosis of toxoplasmosis in each trimester separately and for all trimesters together. Heterogeneity was explored by meta- regression with mixed-effects linear models, where the components of variance were estimated by maximum restricted likelihood. The specified variables for this exploration were the elements used in the risk of bias evaluation by QUADAS 2, sample characteristics used in the original studies, and index test characteristics used in original studies. However, the I^2^- and p-values from Cochrane’s Q test were obtained for all trimesters together, each trimester individually, and early programmed elements that eventually displayed significance in this analysis.

Forest plot graphics were created for each group of data separated by the trimester of pregnancy when the diagnostic investigation occurred.

## Results

### Risk of Bias in the Study

The risk of reporting bias was explored by using funnel charts and Begg tests for each group defined by pregnancy trimester and variables found to be significant during an exploration of the data’s heterogeneity.

### Selection of Studies

**Fig 1:**
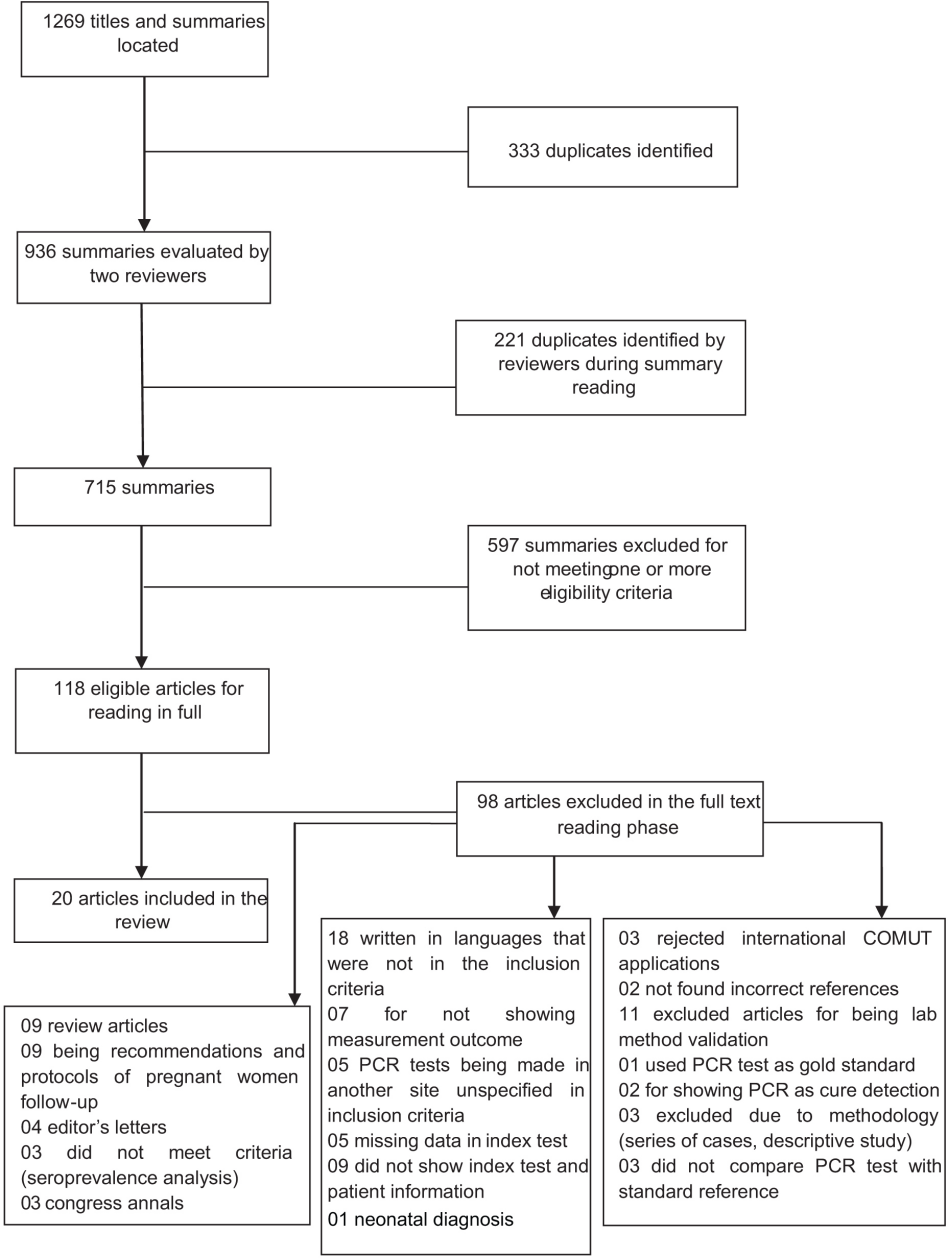
Flowchart of the search results.

The initial search returned 1.269 summaries and titles, 715 of which remained after the exclusion of duplicates. Applying the inclusion and exclusion criteria, 118 summaries were selected to have the full texts recovered. The full texts were evaluated independently by three reviewers. At the end of this phase, 20 articles were selected for inclusion in the review (Fig 1).

### Characteristics of the Included Studies

#### The Sample

The 20 works included [4,6,12,19–35] a total of 4.171 samples (Table 1). Nine (45%) included works that were conducted at multiple centers, and one was a multi-center study with research subjects from many countries, including France, England, Italy, Kuwait, Austria, and Denmark. The locations of the other studies were as follows: 11 (55%) in France, 5 (25%) in Brazil, one (5%) in Austria, one (5%) in Greece, and one (5%) in Norway. Two of the included studies provided data from two or more PCR tests for the same population [33,35]. Only 3 (15%) reported the average age of the pregnant women sampled, which varied between 19 and 28 years. The average pregnancy stage in which the acute maternal infection occurred was provided in three studies (15%) and varied between 15.6 and 19.6 weeks. The period between diagnosis of maternal infection and fetal investigation varied from 3.5 to 9.1 weeks, and these data were reported in ten (50%) studies. There were no reports of HIV-positive pregnant women among the included studies. The evaluation by an ethics and research committee was not explicit in 15 studies (75%). The biological sample used in the 20 included studies (100%) was amniotic fluid.

**Table 1:** Characteristics of included studies.

Author/year	Nationality	Sample size	Multi-center	Average age in years of the pregnant women	Proportion in first trimester	Proportion in second trimester	Proportion in third trimester	Average gestational age in weeks of the acute maternal infection	Time in weeks between maternal diagnosis and fetal investiga tion	Evidence that previous treatment in the pregnant women has influenced the index test	Proportion of pregnant women who received treatment before fetal investigation
Cazenave J (1992)	France	80	No	NI	NI	NI	NI	NI	NI	NI	NI
Dupouy Camet J (1992)	France	44	No	NI	NI	NI	NI	NI	NI	NI	100
Hohlfeld P (1994)	France	339	No	NI	NI	NI	NI	NI	4	NI	100
Jenum PA (1998)	Norway	67	Yes	NI	NI	NI	NI	NI	NI	No	47
Gangneux FR (1999)	France	94	Yes	NI	1.4	36.3	82.7	NI	4	NI	100
Rumand S (2001)	France	270	Yes	NI	23.7	47	29.3	18.3	7	No	96.7
Castro FC (2001)	Brazil	37	No	NI	NI	NI	NI	NI	NI	NI	100
Andrade GMQ (2001)	Brazil	27	No	NI	17.4	25.6	10.5	NI	NI	NI	90.2
Antsaklis A (2002)	Greece	79	No	NI	NI	NI	NI	19.6	4	No	NI
Bessieres MH (2002)	France	261	No	NI	NI	NI	NI	NI	3	NI	100
Vidigal PUT ((2002)	Brazil	86	Yes	19	NI	NI	NI	NI	NI	NI	NI
Thalib L (2005)	Kuwait, France, England, Austria, Denmark	593	Yes	NI	60.2	33.7	6	NI	9	No	NI
Ordinaire I (2005) A	France	27	No	NI	43.3	26.7	30	NI	NI	NI	NI
Ordinaire I (2005) B	France	27	No	NI	43.3	26.7	30	NI	NI	NI	NI
Filho EAF (2005)	Brazil	45	No	23	35.4	45.8	18.8	15.6	NI	NI	NI
Bessieres MH (2009)	France	275	Yes	NI	29.5	41.2	14.1	NI	4	NI	NI
Rabilloud M (2010)	France	481	No	NI	46.4	28.4	25.2	NI	8	NI	NI
Wallon M (2010)	France	261	Yes	NI	41.7	33.4	24.9	NI	8	NI	NI
Sterkers Y (2012)	France	298	Unclear	28	NI	NI	NI	NI	NI	NI	NI
Teixeira LE (2013) A	Brazil	100	Yes	NI	NI	NI	0	NI	NI	NI	100
Teixeira LE (2013) B	Brazil	100	Yes	NI	NI	NI	0	NI	NI	NI	100
Teixeira LE (2013) C	Brazil	100	Yes	NI	NI	NI	0	NI	NI	NI	100
Teixeira LE (2013) D	Brazil	100	Yes	NI	NI	NI	0	NI	NI	NI	100
Prusa AR (2015)	Austria	707	Yes	NI	NI	NI	NI	NI	3	No	61

NI, not informed.

Test performance at first trimester at seroconversion was reported in six studies (30%); in one of these studies [35], two tests were performed with the same population and yielding the identical results. The test performance at second trimester at seroconversion was reported in six studies that included samples from 731 women, in two these studies more than one test was done [33,35]. The performance in third trimester was reported in five studies that included 187 samples.

Maternal treatment before fetal investigation was reported in ten works, covering a total of 1.685 patients; 1.623 (96.3%) received treatment. Neither data nor sufficient discussions regarding the influence of maternal treatment on the sensitivity of PCR tests sing amniotic fluid were provided in these studies.

Three studies proposed a model to limit the probability of fetal infection based on a test that amplifies T. gondii DNA. In a European multi-center study [28], the possibility of a newborn being infected with congenital toxoplasmosis was evaluated based on the pre-test risks (i.e., based on the pregnancy stage at which maternal antibodies were detected) and the PCR result. The study estimated that for a woman who seroconverts during the first trimester of pregnancy, a positive PCR test would increase the risk of having an infected fetus from 9% pre-test to 84% post-test, whereas a negative PCR test would reduce the risk to 3%. If seroconversion occurs at 36 weeks of pregnancy, a positive PCR test would raise the risk of an infected fetus from 73% pre-test to 99% post-test, and a negative result would reduce the risk to 44%. In another study [31], the negative and positive likelihood ratios (PLRs) and the probability of congenital infection were estimated by PCR analysis of amniotic fluid and the IgM and IgA serology results in newborn blood. The risk of fetal infection could be determined by combining the pre-test probability of vertical transmission and the PLR. In a prospective cohort de 344 women who seroconverted for toxoplasmosis during pregnancy, the authors analyzed the results of PCR tests of amniotic fluid, cord blood, and placenta, according to gestational age at maternal infection and inferred pos test predictive values for diagnosis of congenital toxoplasmosis. A positive PCR on amniotic fluid was always associated with a 100% probability of giving birth to an infected child [32].

#### The Test

In all studies, the PCR test was an in-house assay; that is, it was not commercially available. Regarding the type of test, four studies (20%) employed quantitative real-time PCR and 16 (80%) used qualitative PCR, in two of these studies more than one assay was used [33,35]. No two studies used identical protocols for PCR (Table 2).

**Table 2:** Description of the original investigations included regarding the test characteristics.

Author/year	In house test	PCR quantitative/qualitative	Primer	Amplified region	Biological sample used
Cazenave J (1992)	Yes	Qualitative	TG1/TG2/TG3	R-DNA	AF
Dupouy Camet J (1992)	Yes	Qualitative	418–700	P30	AF
Hohlfeld P (1994)	Yes	Qualitative	B22-B23	B1 gene	AF
Jenum PA (1998)	Yes	Qualitative	NI	B1 gene	AF
Gangneux FR (1999)	Yes	Qualitative	*1	B1 gene/TGRE1E	AF
Rumand S (2001)	Yes	Qualitative	NI	B1 gene	AF
Castro FC (2001)	Yes	Qualitative	NI	B1 gene	AF
Andrade GMQ (2001)	Yes	Qualitative	NI	B1 gene	AF
Antsaklis A (2002)	Yes	Qualitative	NI	B1 gene	AF
Bessieres MH (2002)	Yes	Qualitative	694-887/1793-1907	B1 gene	AF
Vidigal PUT (2002)	Yes	Qualitative	OUTER1-OUTER4/INNER2-INNER3	B1 gene	AF
Thalib L (2005)	Yes	Qualitative	NI	NI	AF
Ordinaire I (2005) A	Yes	Qualitative	694–887	B1 gene	AF
Ordinaire I (2005) B	Yes	Quantitative	*2	B1 gene	AF
Filho EAF (2005)	Yes	Qualitative	NI	NI	AF
Bessieres MH (2009)	Yes	Qualitative/Quantitative	*3	B1 gene and RE sequence	AF
Rabilloud M (2010)	Yes	Qualitative	NI	B1 gene	AF
Wallon M (2010)	Yes	Quantitative	NI	529bp	AF
Sterkers Y (2012)	Yes	NI	B22/B23	B1 gene	AF
Teixeira LE (2013) A	Yes	Conventional	JW62-JW63/B22-B23	NI	AF
Teixeira LE (2013) B	Yes	Nested	JW62-JW63/B22-B23	NI	AF
Teixeira LE (2013) C	Yes	Multiplex Nested	JW62-JW63/B22-B23	NI	AF
Teixeira LE (2013) D	Yes	Quantitative	JW62-JW63/B22-B23	NI	AF
Prusa AR (2015)	Yes	NI	NI	B1 gene	AF

*1 primers sequence: B1 5´-CCGCCTCCTTCGTCCGTCGTA and 5´-TGAAGAGGAAACAGGTGGTCG

TGR1E 5´- ATGGTCCGGCCGGTGTATGATATGCGAT and 5´-TCCCTACGTGGTGCCGCAGTTGCCT

*2 primers sequence 5´-CGGAAATAGAAAGCCATGAGGCACTCC and 5´-ACGGGCGAGTAGCACCTGAGGAGAT

*3 primers described in other article (given as reference–Cassaing et al. 2006): B1 5`-GGAGGACTGGCAACCTGGTGTCG and 5´-TTGTTTCACCCGGACCGTTTAGCAG RE 5´-AGGCGAGGGTGAGGATGA and °

AF,amniotic fluid; MB,Maternal blood; NI,not informed

Among the included studies, 15 (75%) amplified the target DNA of the B1 gene. The primers used for the gene amplification process varied among the studies.

All studies used clinical and serological assessments of the infant during the first year of life as a reference. Some works used additional tests for reference, such as inoculation of amniotic fluid in the peritoneum of mice (11 studies, 55%), parasite isolation in cell culture (three studies, 15%), and histopathological exam of the placenta (one study, 5%).

### Results for Methodological Quality Evaluation and Risk of Bias

The low risk of bias was found in the applicability of the test rather than how studies were conducted and reported (Table 3).

**Table 3:** Risk of bias by QUADAS 2.

Author/year	Selection of patients	Index test	Reference test	Flow and time	Selection of patients	Index test	Reference test
Cazenave J (1992)	Unclear	Unclear	Unclear	Unclear	Unclear	Unclear	Unclear
Dupouy Camet J (1992)	Unclear	Unclear	Unclear	Unclear	Low	Low	Low
Hohlfeld P (1994)	Low	Low	Unclear	Low	Low	Low	Low
Jenum PA (1998)	Low	Low	Low	Low	Low	Low	Low
Gangneux FR (1999)	Low	Low	Unclear	Low	Low	Low	Low
Rumand S (2001)	Low	Unclear	Unclear	Low	Low	Low	Low
Castro FC (2001)	Low	Unclear	Low	Low	Low	Low	Low
Andrade GMQ (2001)	Unclear	Unclear	Low	Unclear	Low	Unclear	Low
Antsaklis A (2002)	Low	Unclear	Unclear	Low	Low	Low	Low
Bessieres MH (2002)	Low	Unclear	Low	High	Low	Low	Low
Vidigal PUT (2002)	Unclear	Low	Unclear	Unclear	Low	Low	Low
Thalib L (2005)	Low	High	High	High	Low	Low	Low
Ordinaire I (2005) A	Unclear	Unclear	Low	Unclear	Low	Low	Low
Ordinaire I (2005) B	Unclear	Unclear	Low	Unclear	Low	Low	Low
Filho EAF (2005)	Low	Low	Low	Unclear	Low	Low	Low
Bessieres MH (2009)	Low	High	High	High	Low	Low	Low
Rabilloud M (2010)	Unclear	Unclear	Unclear	Unclear	Low	Low	Unclear
Wallon M (2010)	High	Unclear	Unclear	Low	Low	Low	Low
Sterkers Y (2012)	Unclear	Unclear	Unclear	Low	Low	Low	Low
Teixeira LE (2013) A	Unclear	Unclear	Unclear	Low	Low	Low	Low
Teixeira LE (2013) B	Unclear	Unclear	Unclear	Low	Low	Low	Low
Teixeira LE (2013) C	Unclear	Unclear	Unclear	Low	Low	Low	Low
Teixeira LE (2013) D	Unclear	Unclear	Unclear	Low	Low	Low	Low
Prusa AR (2015)	Low	Unclear	Unclear	Low	Low	Unclear	Low

Low, low risk of bias; high, high risk of bias; Unclear, unclear risk of bias.

**Fig 2:**
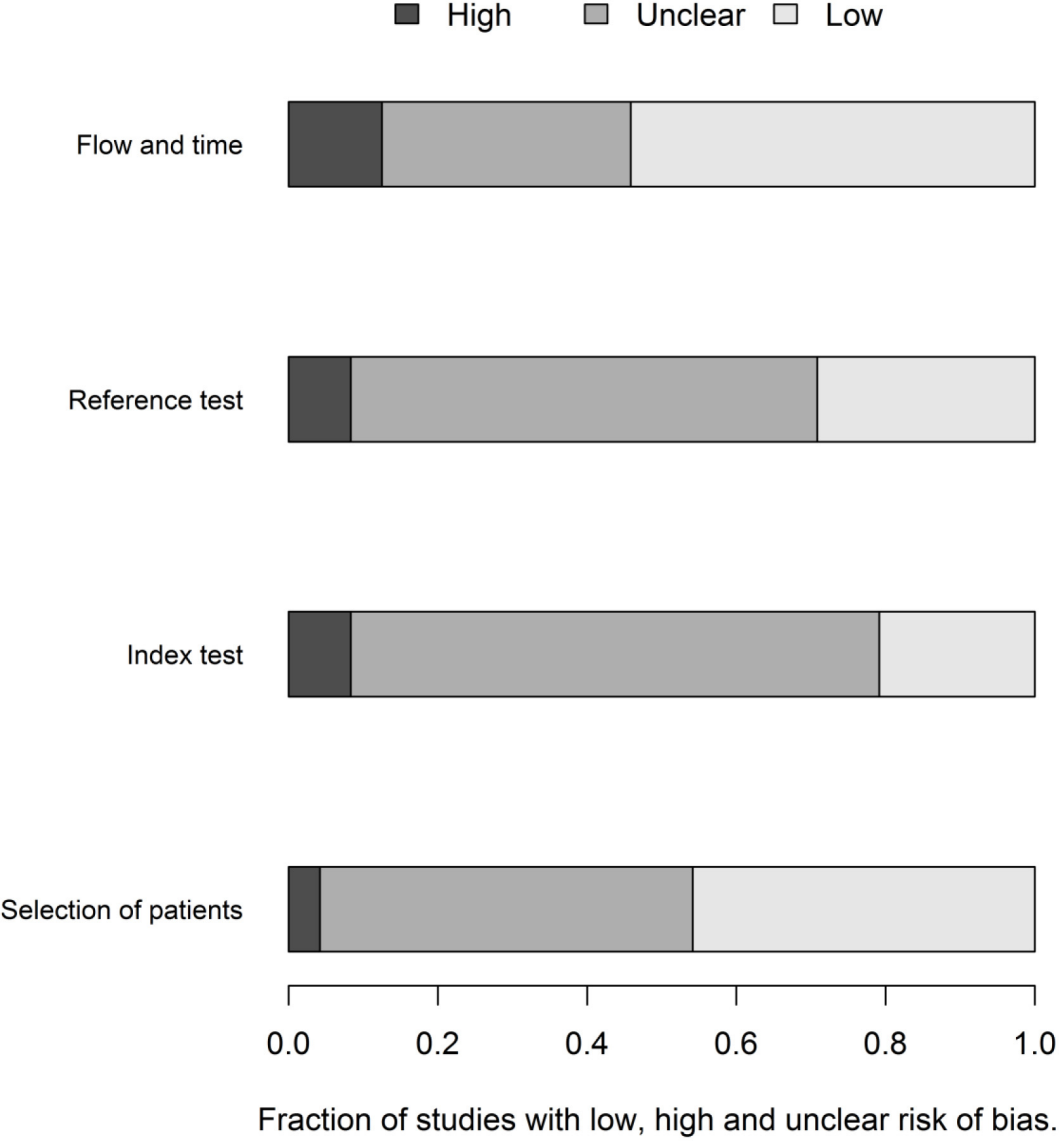
QUADAS 2 –fraction of studies with low, high or unknown risk of bias.

The flow of patients through the study was the domain most susceptible to bias, followed by the conduction of the index and the reference tests. The greatest rate of “unclear” results was in relation to the index test, followed by the reference test. (Fig 2).

**Fig 3:**
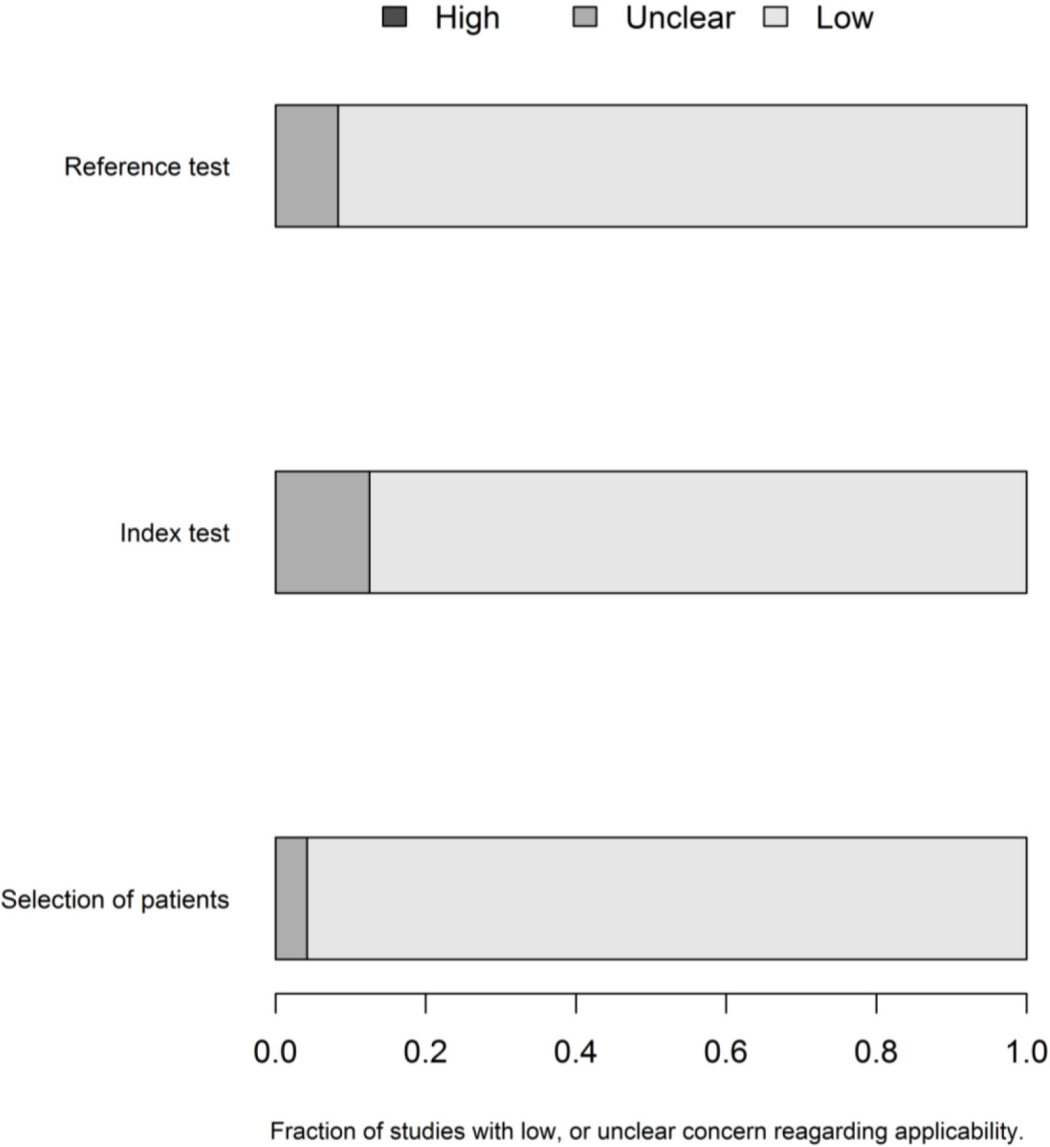
QUADAS 2 –applicability.

The lack of information regarding the risk of bias domains makes it difficult to truly appreciate the quality of research conduction reporting. Regarding the applicability, the risk of bias was lower and was similar in the three domains (Fig 3).

### Results of Individual Studies and Synthesis of the Results for All Trimesters

#### Heterogeneity and Performance for All Trimesters

There is strong evidence of heterogeneity in this meta-analysis, although it is concentrated in the test sensitivity. There was no evidence that stratifying the test performance by pregnancy trimester would reduce the heterogeneity in sensitivity. In contrast, the low heterogeneity found in specificity and DOR were substantially reduced in these subsets, although the reduced sample size in each trimester may impede the interpretation of this result. No correlation between the false positive rate and sensitivity of any subgroup was observed, indicating that the detection limit was not a source of heterogeneity. However, false positive results could be attributed to DNA contamination during PCR assay. The global sensitivity of the PCR test was 83%, although there was a high level of heterogeneity (I^2^ = 66.5%). The global specificity of the test was 98.3%. The DOR for all trimesters together was 453.482, with moderate evidence of heterogeneity (Table 4).

**Table 4:** PCR performance for fetal Toxoplasmosis diagnosis for all the trimesters and each trimester of pregnancy.

Group/statistics	Model	Estimate	CIinf	CIsup	I2	CIinf	CIsup
All studies(n = 24)	-	-	-	-	-	-	-
Se	Bivariate	0.83	0.771	0.877	0.665	0.486	0.782
Sp	Bivariate	0.983	0.969	0.991	0.688	0.525	0.795
DOR	D&L	453.482	182.933	1124.155	0.484	0.171	0.679
Correlation	-	-0.054	-0.447	0.357	-	-	-
1° Tri(n = 6)	-	-	-	-	-	-	-
Se	Bivariate	0.569	0.395	0.727	0	0	0.659
Sp	Bivariate	0.992	0.982	0.997	0	0	0
DOR	D&L	305.289	66.95	1392.102	0	0	0.214
Correlation	-	0.589	-0.426	0.948	-	-	-
2° Tri(n = 10)	-	-	-	-	-	-	-
Se	Bivariate	0.879	0.782	0.936	0.698	0.419	0.843
Sp	Bivariate	0.964	0.938	0.98	0.191	0	0.598
DOR	D&L	269.219	107.73	672.781	0.139	0	0.551
Correlation	-	-0.43	-0.834	0.273	-	-	-
3° Tri(n = 6)	-	-	-	-	-	-	-
Se	Bivariate	0.756	0.66	0.831	0	0	0.717
Sp	Bivariate	0.912	0.802	0.963	0	0	0.156
DOR	D&L	55.552	9.89	312.037	0	0	0.727
Correlation	-	-0.173	-0.863	0.743	-	-	-

CIinf, 95% confidence interval inferior limit; CIsup, 95% confidence interval superior limit; DOR, diagnostic odds ratio; I^2^, heterogeneity statistic; n, number of studies, Se, sensitivity, Sp, specificity

There is evidence that the test sensitivity varies by trimester, increasing from the first to third trimester. There was also a progressive reduction in DOR and specificity from the first to third trimester, although this reduction was less extreme than the reduction in sensitivity. However, these observations lack statistical significance and most likely suffer from limitations due to heterogeneity.

The mean sensitivity of the test during the first trimester of pregnancy was 56.9%. The mean specificity in the first trimester was 99.2%. The DOR estimate for the first trimester was 305.2. The sensitivity estimate for the second trimester was 87.9%. The specificity estimate in the third trimester was of 91.2%, representing a slight decrease compared to the two previous trimesters. The DOR estimate for the third trimester was 55.5 (Table 4).

Another characteristic that was explored to reduce heterogeneity was the time between maternal investigation and fetal investigation. Accounting for characteristic did reduce heterogeneity; however, it was explored for all trimesters combined because there were an insufficient number of studies in each individual stage.

### Sensitivity, Specificity, and DOR in All Trimesters According to Time Between maternal Diagnosis and Fetal Investigation

Test specificity varied only slightly among fetal tests performed more than five weeks after maternal diagnosis compared to those performed less than five weeks after maternal diagnosis. However, the sensitivity was higher when there was less time between maternal diagnosis and fetal investigation (Table 5).

**Table 5:** PCR performance for fetal Toxoplasmosis diagnosis for the different periods in maternal and fetal investigation.

Group/statistics	Model	Estimate	CIinf	CIsup	I2	CIinf	CIsup
<5 weeks(n = 6)	-	-	-	-	-	-	-
Se	Bivariate	0.872	0.821	0.911	0.156	0	0.786
Sp	Bivariate	0.994	0.987	0.997	0	0	0.539
DOR	D&L	1739.391	592.778	5103.907	0	0	0.483
Correlation	-	-0.27	-0.887	0.694	-	-	-
≥5 weeks(n = 4)	-	-	-	-	-	-	-
Se	Bivariate	0.738	0.599	0.841	0.698	0.13	0.895
Sp	Bivariate	0.99	0.98	0.995	0	0	0.831
DOR	D&L	281.232	84.803	932.649	0.392	0	0.793
Correlation	-	-0.308	-0.979	0.928	-	-	-

CIinf, 95% confidence interval inferior limit; ICsup, 95% confidence interval suprior limit; DOR, diagnostic odds ratio; D&L, DerSimonian & Laird; I^2^, heterogeneity statisctic; n, number of studies, Se, sensitivity, Sp, specificity.

Publishing bias did not appear to be a concern. When there was evidence of publishing bias, it occurred in analyses of groups with fewer than 10 studies and with considerable evidence of heterogeneity, which rendered these results invalid.

**Fig 4:**
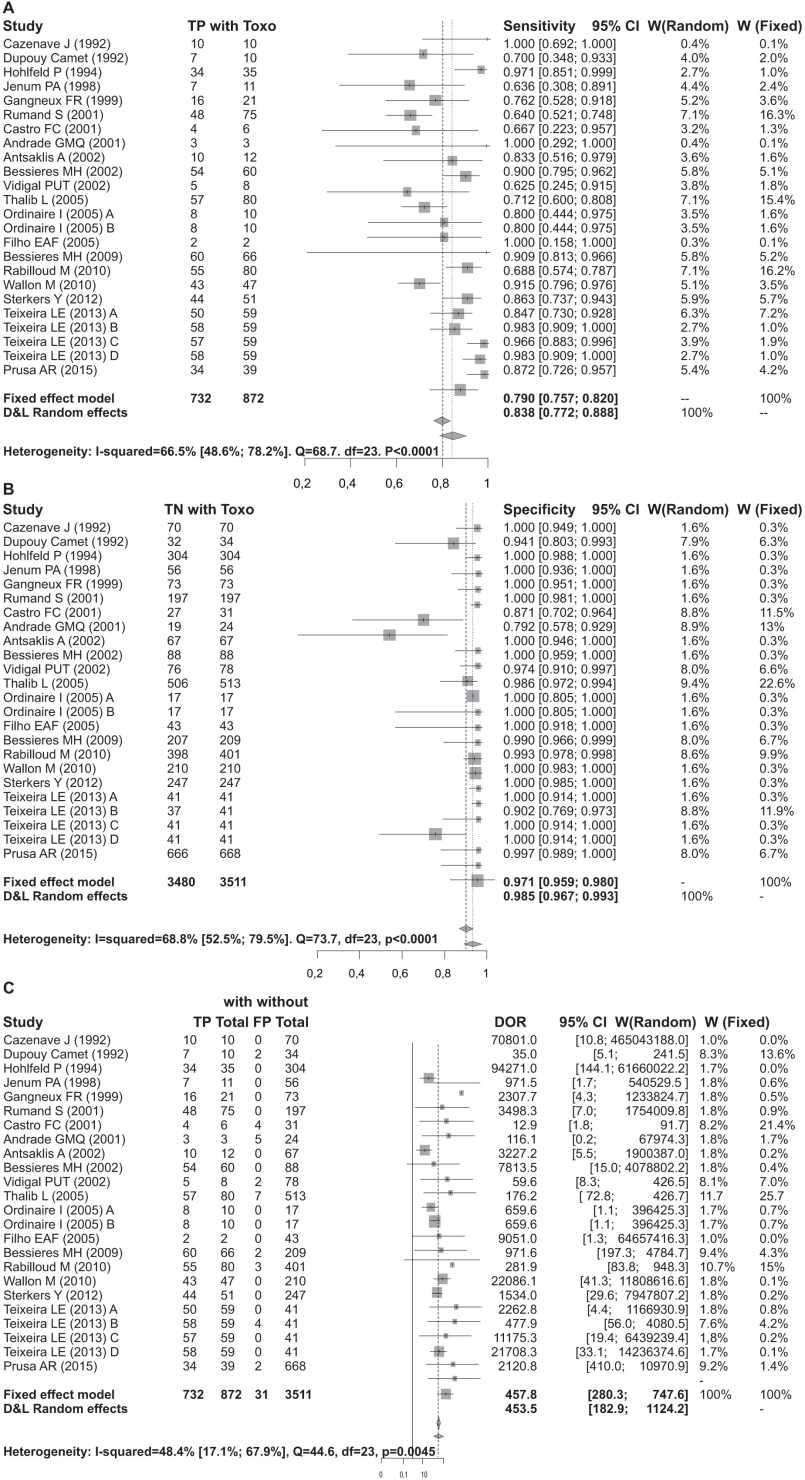
Forest for sensitivity, specificity and diagnostic odds ratio for all trimesters.

**Fig 5:**
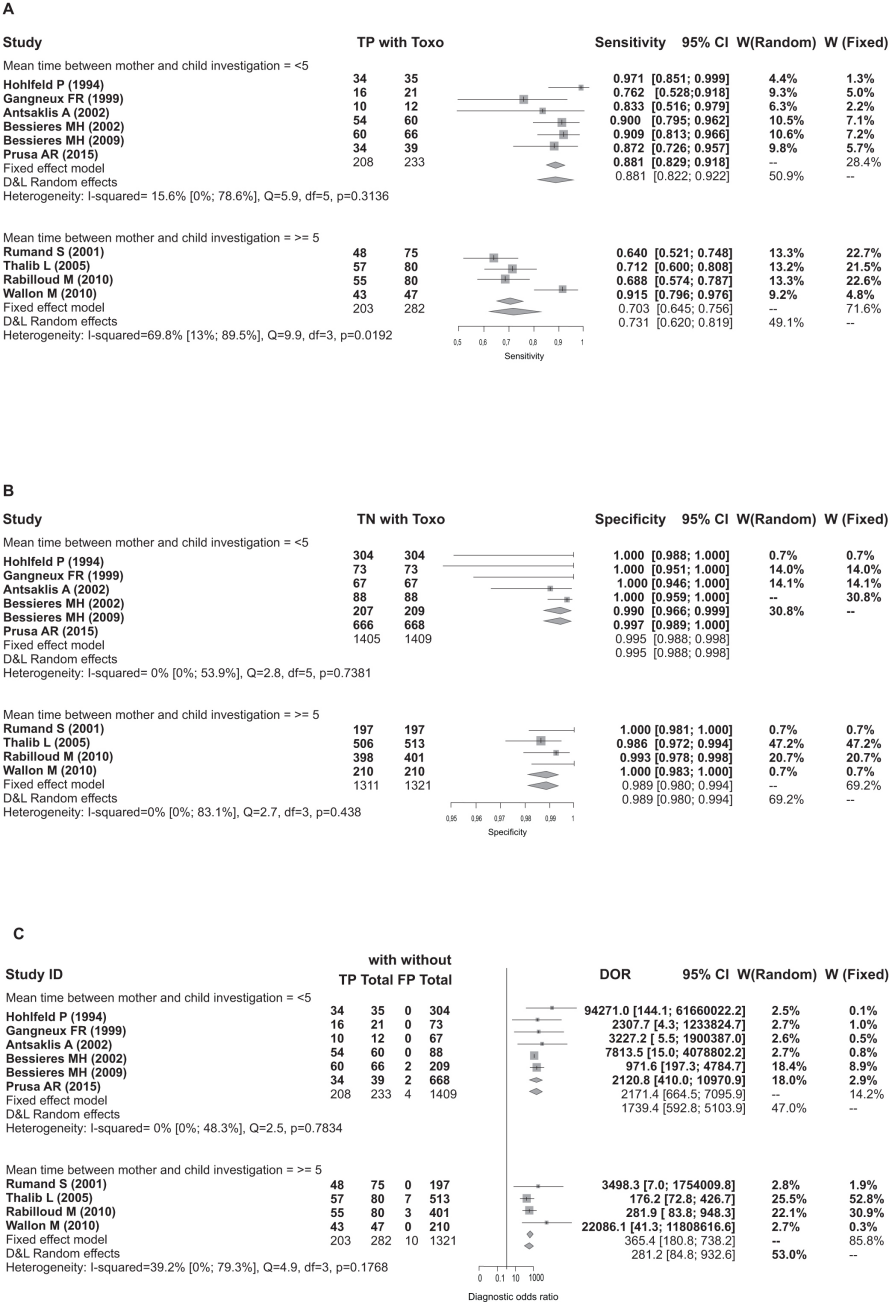
Forest Sensitivity, Specificity and DOR all trimesters for investigation time.

**Fig 6:**
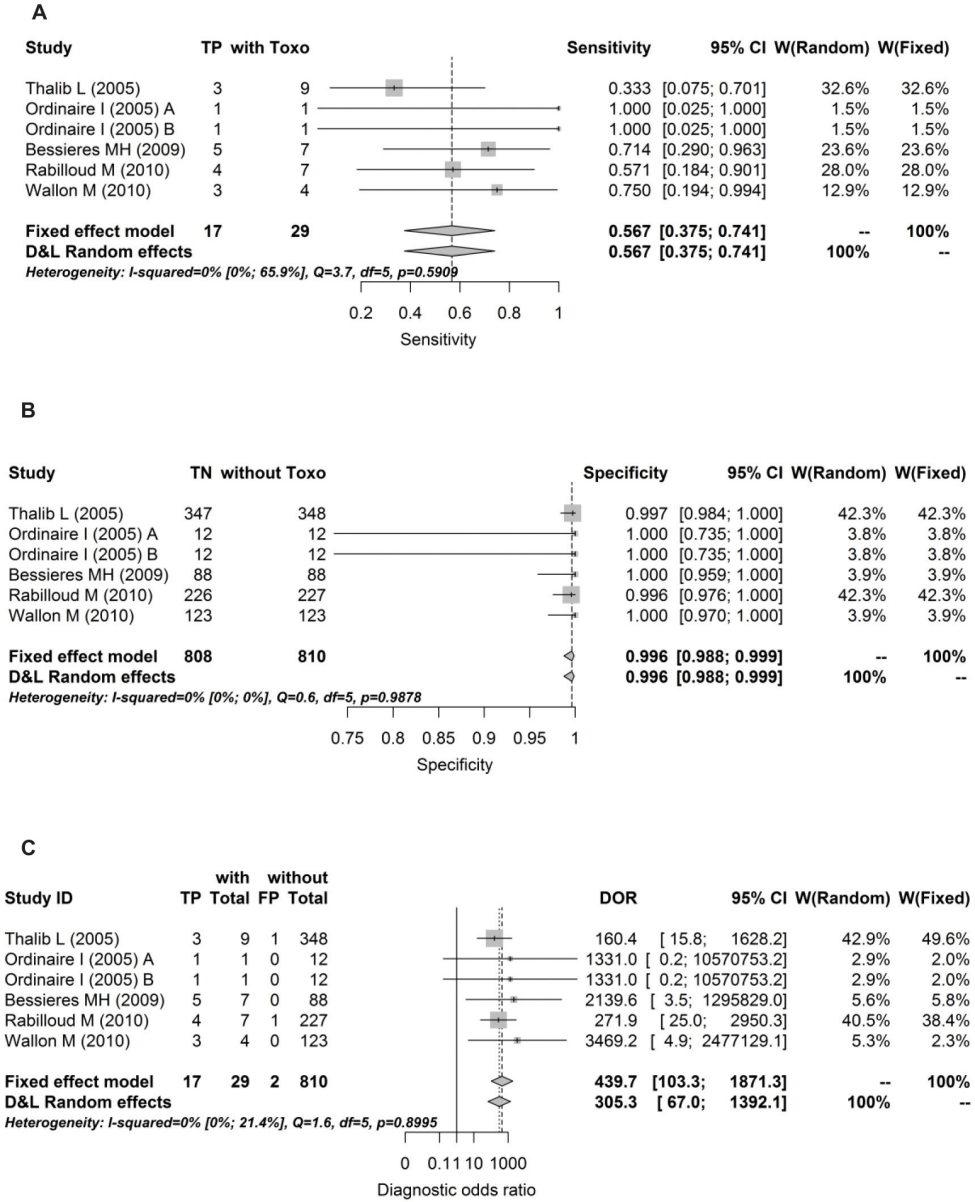
Forest for sensitivity, specificity and diagnostic odds ratio for 1^st^ trimesters.

**Fig 7:**
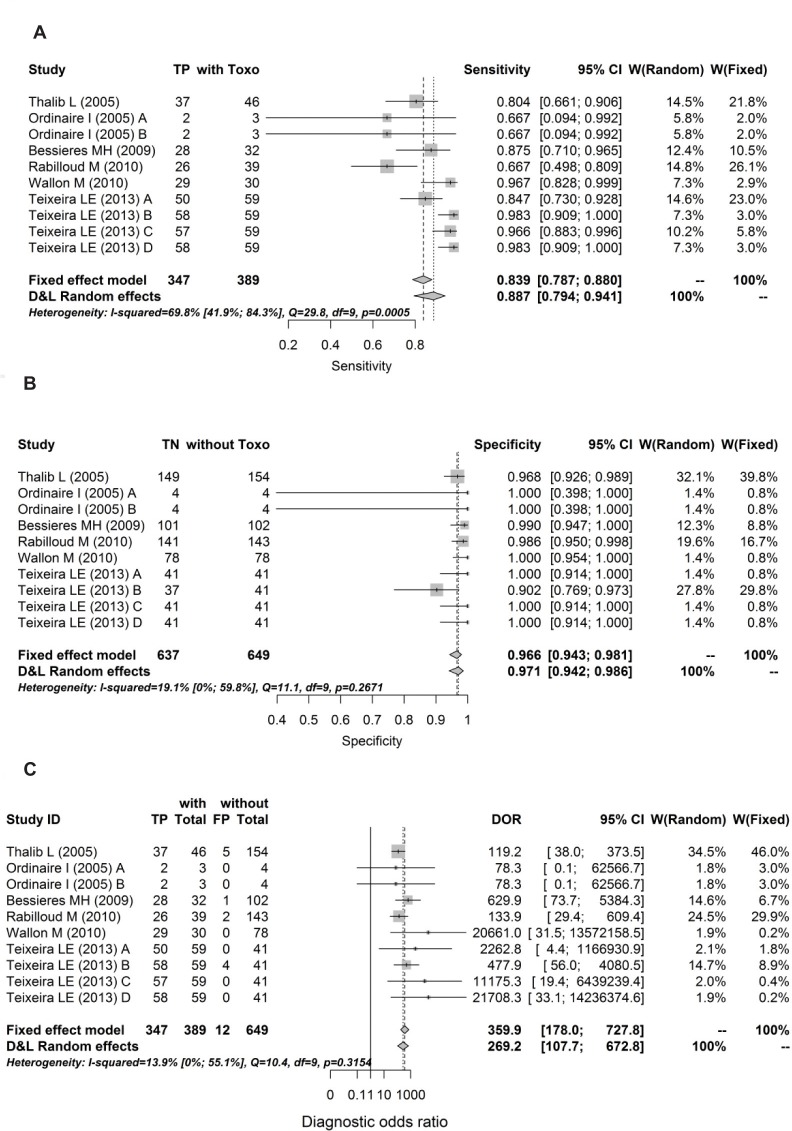
Forest for sensitivity, specificity and diagnostic odds ratio for 2^nd^ trimesters.

**Fig 8:**
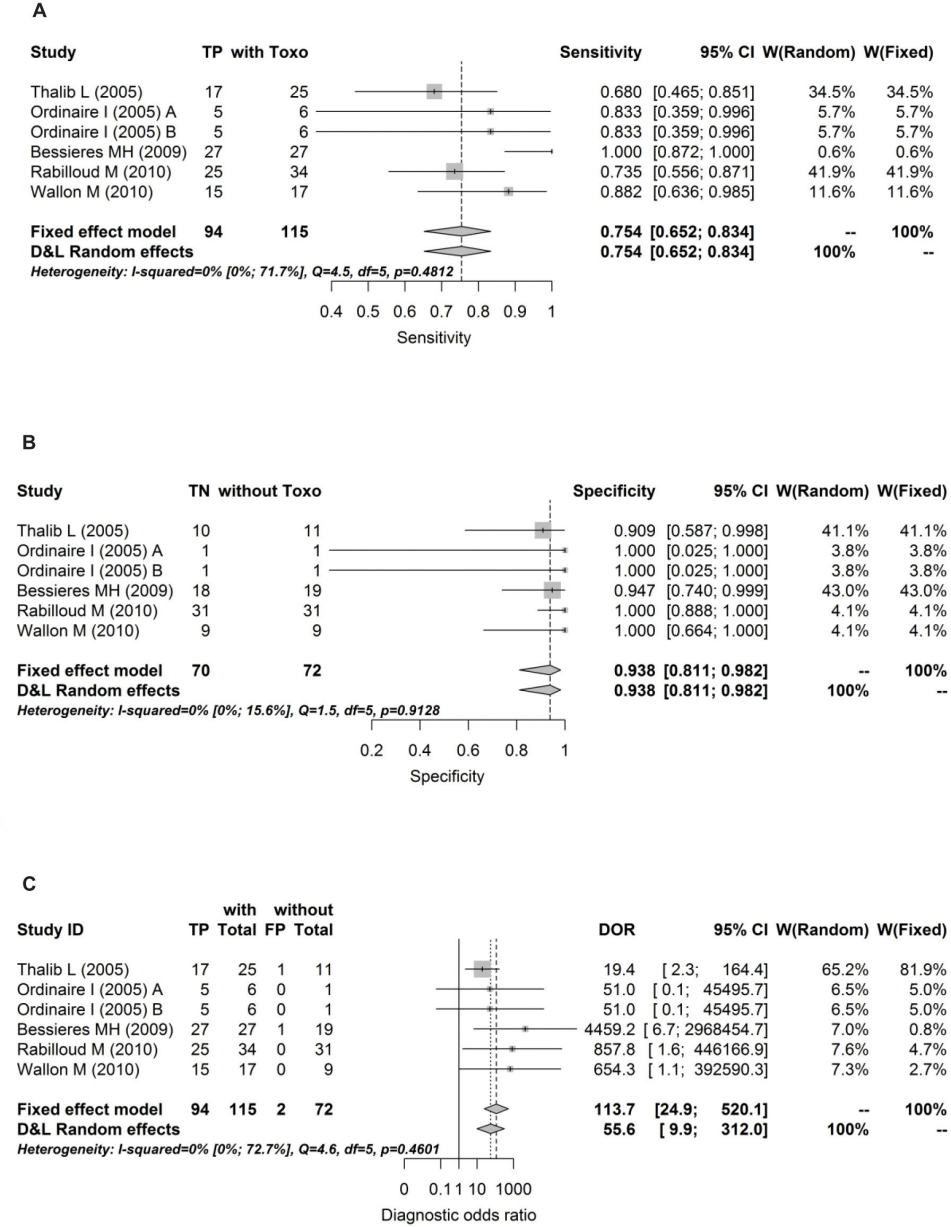
Forest for sensitivity, specificity and diagnostic odds ratio for 3^rd^ trimesters.

Forrests are presented below in Figs 4, 5, 6, 7 and 8.

## Discussion

The diagnosis of congenital toxoplasmosis can be difficult, combining clinical features and results from a battery of serologic and molecular test in the prenatal, neonatal, and postnatal periods [32].

Although the efficacy of prenatal treatment is debatable, it is always recommended whenever there is a serological suspicion of maternal infection during pregnancy [36]. The early detection and treatment of congenital toxoplasmosis may be able to reduce the risk of severe clinical lesions [37]. Prenatal treatment within four weeks of seroconversion was found to reduce the risk of intracranial lesions compared to no treatment, and a delay of more than eight weeks between maternal seroconversion and the beginning of treatment was associated with an increased risk of retinochoroiditis during the first two years of life in infected children [38]. In a study in Brazil, when patients were treated with spiramycin during pregnancy, there was protection against severe form of congenital infection [39]. In a systematic review [40] performed to evaluate the efficacy of treatment for congenital toxoplasmosis during pregnancy, an association was observed between early treatment and a reduction in the risk of congenital toxoplasmosis. Therefore, a diagnostic method capable of identifying cases that merit treatment is essential.

The main results of this review are as follows: (a) the utility of PCR tests for the diagnosis of fetal toxoplasmosis is poorly studied, and reports on this topic lack information, which complicates the interpretation of the results; (b) the protocols vary among studies; (c) the heterogeneity is concentrated in the sensitivity; (d) there is evidence that the test sensitivity increases with progressive trimesters; and (e) there is evidence that the heterogeneity is concentrated in studies in which there was more time between maternal diagnosis and fetal investigation.

In our review and evaluation by QUADAS 2, we found that the risk of bias was primarily related to the study methodology, particularly the choice of the index and reference tests. We observed no reduction in the risk of bias with publication time; the risk was similar between the older and more recent studies, reflecting the lack of significant advancements in study methodology and reporting in this field despite initiatives, such as Standards for Reporting of Diagnostic Accuracy (STARD) [41]. Variability in the protocols of primary studies was observed with different DNA extraction methods, different amplification targets, and variability in PCR primers. Such differences may be the source of inter-laboratory variation in trial performance, making it difficult to compare data from the various centers that perform this test. In a survey of laboratory practices, a similar diversity in execution protocols for PCR tests for toxoplasmosis was described [7]. There is no recent discussion of how the differences among protocols influences test performance; however, test performance can vary due to more than simply laboratory quality or proficiency. As there are no available data about the reliability for these tests, the variation in performance may be due to intrinsic variation in methodologies or operator dependency, in other words, low reproducibility. Such problems are not exclusive to assays for toxoplasmosis diagnosis but also exist for any molecular test for parasites, bacteria, or other infectious agents with complex genetic behavior and characteristics. These problems could be minimized if a commercial test were widely available.

The existence of many protocols may be related to two fundamental aspects: (a) adaptations of an original protocol to situations that change over time and in different places according to available resources and (b) the perception that the tests can be improved through protocol modifications.

We observed that the heterogeneity is concentrated in the sensitivity; the sensitivities vary so widely that they likely do not correspond to the same test. The statistical measure of heterogeneity can represent differences in tests, samples, or investigation procedures.

Heterogeneity is often attributed to varying test protocols derived from the lack of a standardized method. The experience and technical ability, quality, and proficiency control of each laboratory are fundamental elements necessary to solve this problem [7].

Although the heterogeneity in this investigation was not completely explained, there was a considerable reduction in the heterogeneity for sensitivity when the results were stratified by the time between maternal diagnosis and fetal investigation. This result indicates that beyond the differences among test protocols, the clinical protocols and disease development during this period may be primarily responsible for the observed differences in test performance.

Relevant events that may occur between maternal diagnosis and fetal investigation include, for instance, variations in parasitaemia. It is reasonable to imagine an increasing initial parasitaemia followed by a peak and a period of decreasing parasitaemia. However, this behavior is based on anecdotes and thus speculative; rigorous evidence remains nonexistent. Tests performed on samples collected during these different periods of infection can simplify or complicate the DNA amplification of the parasite depending on the initial amount of parasite in the sample. Additionally, the encystment of the parasites can occur in unfavorable conditions, which in turn complicates DNA identification.

It has previously been suggested that the treatment be instituted up to three weeks after maternal seroconversion, with the intent of reducing transmission of the maternal infection to the fetus [42]. However, some therapeutic schemes used to treat the maternal infection would also affect the fetal infection. From a clinical perspective, beginning fetal infection treatment before fetal diagnosis can interfere with the test’s performance, but this interference would be homogeneous if the proposed treatment were implemented uniformly. As there was no information about treatment in most of the investigations included in this review, the beginning of a therapeutic regime may have interfered with the severity of the infection and therefore the test’s capacity to identify infected subjects. However in a cohort de 707 cases of maternal acute infection, where 432 women received amniocentesis after starting treatment, this did not influence sensitivity or specificity of the PCR [34].

Our review provided evidence that the test’s sensitivity increases with pregnancy trimester, although there were no statistically significant differences. This observation is based on data in the literature [31]. The lower sensitivity of the test in the first trimester can be explained by low concentrations of fetal cells in the amniotic fluid during the first trimester or by infections with low parasite load in the amniotic fluid [5,7,28], likely due to the low permeability of the placenta in the first trimester.

Even though the possible explanations for this observation are speculative, if confirmed, this observation is relevant to the diagnosis of fetal toxoplasmosis; similar results from tests conducted during different stages of pregnancy could have different interpretations.

Another result from our review is that heterogeneity tends to be concentrated in studies where there was more time between maternal diagnosis and fetal investigation. Test performance tended to be better for investigations performed up to five weeks after the diagnosis of acute maternal infection compared to investigations performed after this period. The literature recommends that amniocentesis should not be offered for the identification of *Toxoplasma gondii* infection at less than 18 weeks’ gestation and should be offered no less than 4 weeks after suspected acute maternal infection to lower the occurrence of false-negative results [43,44].

Although there is a lack of evidence evaluating the effect of treatment on fetal infection, a possible interpretation is that maternal treatment may affect test performance; in other words, when the treatment is instituted before conducting a diagnostic investigation, the parasite load may be reduced such that the identification of the parasite is hindered. Authors seldom discuss possible interference of the treatment with the test when treatment is started before the diagnostic investigation, and the few authors who do comment on this matter did not observe this effect [13,22,34]. In any case, from a clinical perspective, it would be difficult to justify the institution of a delay in treatment to wait for a diagnosis given the potential consequences. Thus, all tests performed in practice will suffer from interference due to the effects of treatment. Therefore, it is important to study the magnitude of this interference.

A pertinent hypothesis suggested previously [28] is that the maternal and fetal immune responses might reduce the amount of free parasite over time, decreasing the PCR test’s sensitivity with an increasing interval between maternal seroconversion and amniocentesis.

A limitation of this study was the exclusion of 17 articles available in languages inaccessible to the reviewers [45–61] and the exclusion of three articles because they were archived in international libraries and copies were not allowed [62–64]. The absence of these studies could modify the estimated sensitivity and specificity obtained in this analysis. However, based on reading the summaries, the absence of these works is unlikely to have considerable influence because most included sample sizes of a few dozen. It is therefore unlikely that the heterogeneity in sensitivity would have been reduced with the inclusion of these works.

As discussed above, our inability to fully explain the heterogeneity is a limitation of this work. The presence of heterogeneity means that the estimated sensitivity does not represent all included tests, which makes the test’s overall sensitivity unknown. Although we have found that heterogeneity decreases in studies in which the average of time between maternal diagnosis and fetal investigation was less than five weeks, these data were not available for many of the included studies. Additionally, we must consider that this information is aggregated by study and does not consider each patient individually. Besides this, we haven´t been able to identify if contamination in DNA assay could be responsible for results different of 100% in specificity before or after 5 weeks of maternal infection.

It was possible to ensure that none of the diagnostic tests related a reproducibility measure. Noncommercial diagnostic tests and tests that are highly operator dependent typically have a higher variability in their measurements, that is, a lower reproducibility. A fundamental question of reproducibility is how variable the measures can be for the tests to be considered reproducible. This concept is not directly connected to test validity but does interfere with the interpretation of its results. Some authors have adopted more automated procedures [4,35,30] and have argued that the lower degree of operator dependence improves test reliability; however, they did not report any measure of reliability. All systematic reviews are influenced by the quality of the original studies included. When studies that lack desired qualities are included, the conclusions are limited. However, the evidence is still relevant because it demonstrates that current research practices are based on insufficient evidence and suggests ways to refine data collection.

Despite the limitations regarding the diagnostic performance of PCR analysis of amniotic fluid, this method provides operational advantages compared to other methods, such as mouse inoculation. PCR analysis of amniotic fluid is the fastest method, yielding results within 4 h, and it is the safest method, with a fetal loss rate attributed to amniocentesis of 0.5–1% [43]. Despite the problems with PCR sensitivity identified in this investigation, this test is still the most sensitive for prenatal congenital toxoplasmosis diagnosis when compared to culture, mouse inoculation, and fetal blood serology [11].

Clinical and serological follow-up of the infant is the most accepted and most widely used method of determining a definitive diagnosis of congenital toxoplasmosis and discarding false negative or false positive molecular diagnoses in clinical studies. However, it is reasonable to consider that such follow-up procedures can suffer from the effect of anti-parasite treatment, meaning that correct identification of the parasite followed by treatment can result in the absence of antibodies at the end of successful treatment, thus leading to improper classification of the reference test. The authors of the included texts did not discuss this issue as a possible limitation, although in theory, it would reduce the sensitivity and specificity of the test. Unfortunately, this issue is a possible limitation of any study that uses a follow-up as a reference.

Despite these limitations, the available evidence demonstrates that the PCR test has a sensitivity of 87% and a specificity of 99% when performed up to five weeks after maternal diagnosis. Additionally, the test performance can be differentiated according to pregnancy trimester. Therefore, the test can be recommended in a period when there is increased suspicion of fetal toxoplasmosis. However, the absence of a Kit commercial widely available and reliable, will require health professionals to refer these patients to a research unit capable of executing the test or to treat the suspected infection empirically without diagnosis.

The following aspects must be improved for future investigations of PCR performance for the diagnosis of fetal toxoplasmosis: (a) adherence of research reports to recommended standards; (b) discussion of the extent to which the therapeutic schemes administered before diagnostic investigation impact the test performance; (c) estimation of reliability, even with the use of automated methods; (d) exploration of new targets for amplification that are more repetitive in the *Toxoplasma* DNA; and (e) exploration of how the molecular test results can be combined with other clinical information, particularly the stage of pregnancy at which maternal infection occurred.

For the diagnosis of fetal toxoplasmosis, having PCR as a tool is useful because molecular technologies are becoming less expensive and simpler to execute. Additionally, these technologies are operationally simpler than the alternative tests, and the results can modify the therapeutic regimes for pregnant women identified with acute infection.

We find a lot of heterogeneity in our results. Considering the diversity of parasite concentration in amniotic liquid during the pregnancy, and amniocentesis as an invasive procedure, someone could argue that introduction of monthly prenatal serological screening would be enough to detect seroconversion in appropriate time and then help for a significant reduction in the rate of congenital infection. Maybe a monthly screening using serological and molecular tests could be more suitable for the Brazilian Health System. However this does not invalidate the need for fetal research and its importance for a proper fetal and neonatal approach.

## Conclusion

This review found that the PCR test had a global sensitivity of 83% and specificity of 98.3%; however, these measurements display strong evidence of heterogeneity (I^2^ = 66.5% for sensitivity), which makes difficult to interpret these summary estimates. In the subgroup with an average time between maternal infection and fetal investigation of up to five weeks, there was moderate to low evidence of heterogeneity in sensitivity and specificity (87.2% and 99.4%, respectively).

There was no identical protocol described in the literature, and we were not able to find any data from commercial tests among the studies included in the review. We were able to identify the main limitations and strengths of the literature on this topic, providing insights for future studies on this technique and its clinical implementation.

## Supporting Information

S1 Text Search terms in electronic databases. (PDF)

S2 Text Assessment of selected studies in the full-text stage. (PDF)
