# Supplementary material for: Performance of Polymerase Chain Reaction Analysis of the Amniotic Fluid of Pregnant Women for Diagnosis of Congenital Toxoplasmosis: A Systematic Review and Meta-Analysis
Source: PLoS One. 2016 Apr 7;11(4):e0149938. doi: 10.1371/journal.pone.0149938 (PMC4824461; doi:10.1371/journal.pone.0149938)
Supplement: S1 Text — (PDF) [file pone.0149938.s001.pdf]

S1- SEARCH TERMS IN ELECTRONIC DATABASES

PUBMED

(Diagnosis/Broad[filter]) AND (("pcr"[All Fields]) OR "polymerase chain reaction"[All Fields] OR "polymerase chain reaction"[MeSH] OR (molecular AND test*) AND (toxoplasm* OR "toxoplasmosis" [MeSH]) AND (congenital OR fetal OR prenatal))

SCOPUS

((KEY ("sensitivity and specificity"))OR(TITLE-ABS-KEY(sensitiv*)) OR (TITLE-ABSKEY (diagnos*)) OR (KEY (diagnostic)) OR(KEY (diagnosis))) AND (TITLE-ABS-KEY(PCR)) OR (TITLE-ABS-KEY("polymerase chain reaction")) OR TITLE-ABS-KEY (molecular AND test))) AND (TITLE-ABS-KEY (toxoplasm*)) AND (TITLE-ABS-KEY (congenital OR fetal OR prenatal)))

WEB OF KNOWLEDGE

(((TS=(diagnostic*)) OR (TS=(diagnosis)) OR (TS=(diagnosis)) OR (TS=("sensitivity and specificity")) OR TI=(sensitiv*))) AND ((TS=("molecular test") OR TS=("polymerase chain reaction") OR TS=(pcr)) AND (TS=(toxoplasmosis) OR TS=(toxoplasm*)) AND (TS=(prenatal) OR TS=(fetal) OR TS=(congenital)))

LILACS

("sensitivity and specificity" OR "sensibilidade e especificidade" OR sensi* R diagnostic* OR diagnos*) AND (pcr OR "teste molecular" OR "molecular test" OR "reação em cadeia da polimerase" OR "polymerase chain reaction") AND (toxoplasm*) AND (pre-natal OR prenatal OR "pre natal" OR congenito OR congenital OR fetal)
