# Supplementary material for: Performance of Polymerase Chain Reaction Analysis of the Amniotic Fluid of Pregnant Women for Diagnosis of Congenital Toxoplasmosis: A Systematic Review and Meta-Analysis
Source: PLoS One. 2016 Apr 7;11(4):e0149938. doi: 10.1371/journal.pone.0149938 (PMC4824461; doi:10.1371/journal.pone.0149938)
Supplement: S2 Text — (PDF) [file pone.0149938.s002.pdf]

S2- Assessment of selected studies in the full-text stage.

Systematic review of PCR performance in amniotic fluid, for congenital toxoplasmosis diagnosis

Section 1: Identifying text		
1. Identifier number:	2. Name of reviewer:	
3. Date of review:	4. First author's name:	
5.Year of publication:	6. Journal:	
7. Volume:	8. Number:	9. Page (first):

Section 2: Elegibility full text		
10. This study approaches women who have suspected acute toxoplasmosis during pregnancy?	**1.**Yes **0.**No **-7.**No information	P 10:
11. This study evaluates the accuracy of PCR in amniotic liquid?	**1.**Yes **0.**No **-7.**No information	P 11:
12. This study evaluates the accuracy of PCR in cord blood?	**1.**Yes **0.**No **-7.**No information	P 12:
13. This study evaluates the accuracy of PCR in maternal blood?	**1.**Yes **0.**No **-7.**No information	P 13:
14. This study presentes the outcome measures, sensitivity, specificity, predictive values?	**1.**Yes **0.**No **-7.**No information	P 14:
15. This study compares the results of the PCR test with a reference test?	**1.**Yes **0.**No **-7.**No information	P 15:
16. This study is written in Portuguese, English, French, Spanish or Italian?	**1.**Yes **0.**No **-7.**No information	P 16:
17. This study was included for data extraction from full text?	**1.** Yes **0.**No	P 17:

Section 3: assessment of risk of bias by QUADAS 2		
Regarding the selection of patients:		
**A. Risk of bias**		
18. The sample of patients was consecutive or random?	**1.**Yes **0.**No **9.**It is not clear	P 18:
19. A case-control design was avoided?	**1.** Yes **0.**No **9.**It is not clear	P 19:
20. The study avoided inappropriate exclusions?	**1.** Yes **0.**No **9.**It is not clear	P 20:
21. What is the risk of selection of patients have introduced bias?	**1.** Yes **0.**No **9.**It is not clear	P 21:
**B. Concern about the applicability**		
22. There is concern that the patients included did not correspond to the review question?	**1.**High **0.**Low **9.**It is not clear	P 22:
As to the test index		
**A. Risk of bias**		
23. The resulto f the index test was interpreted without knowledge of the results of the reference test?	**1.** Yes **0.**No **9.**It is not clear	P 23:
24. If a decision threshold (cutoff) was used, it was pre-specified?	**1.** Yes **0.**No **9.**It is not clear	P 24:
25. What is the risk of the conduct or interpretation of the index test may have introduced bias?	**1.** Yes **0.**No **9.**It is not clear	P 25:
**B. Concern about the applicability**		
26. There is concern that the index test, your driving, your interpretation is diferente from review of the matter?	**1.**High **0.** Low **9.**It is not clear	P 26:
As for the reference test		
**A. Risk of bias**		
27. The standard reference test correctly classifies the condition of interest?	**1.** Yes **0.**No **9.**It is not clear	P 27:
28. The results of the reference test was interpreted without knowledge of the index test result?	**1.** Yes **0.**No **9.**It is not clear	P 28:
29. What is the risk of the conduct or interpretation of the reference test have introduced bias?	**1.**High **0.**Low **9.**It is not clear	P 29:
**B. Concern as to the applicability**		
30. There is concern that the condition of interest, as defined by the reference test does not correspond to question of the revision?	**1.**High **0.**Low **9.**It is not clear	P 30:
As the flow of patients and time		

A. RisK of bias		
31. There was an appropriate time interval between the index test and reference?	**1.** Yes **0.**No **9.**It is not clear	P 31:
32. All patients underwent a test reference?	**1.** Yes **0.**No **9.**It is not clear	P 32:
33. All patients were subjected to the same test reference?	**1.** Yes **0.**No **9.**It is not clear	P 33:
34. All patients were included in the analisys?	**1.** Yes **0.**No **9.**It is not clear	P 34:
35. What is the risk of patient flow have introduced bias?	**1.**High **0.**Low **9.**It is not clear	P 35:

Section 4: Characteristics of the study or sample		
36. This study is a multicenter?	**1.** Yes **0.**No **9.**It is not clear	P 36:
37. Nationality of volunteers or country where the research was conducted:	P 37:	
38. Average or median age of the sample of voluntary pregnant?	P 38:	
39a. Proportion of pregnant women in the first trimester diagnosis of acute maternal toxoplasmosis:	P 39a:	
39b. Proportion of pregnant women in the second trimester diagnosis of acute maternal toxoplasmosis:	P 39b:	
39c Proportion of pregnant women in the third trimester diagnosis of acute maternal toxoplasmosis:	P 39c:	
40. What is the average gestational age of acute maternal infection, in weeks?	P 40d:	
41. Was explicit assessment of research by research ethics committee?	**1.** Yes **0.**No **-7.**No information	P 41:
42. Mean / Median time in weeks between diagnosis of maternal infection and fetal research:	P 42:	
43. Proportion of pregnant women who received treatment for toxoplasmosis before fetal research:	P 43:	
44. Average time / average between start of treatment for toxoplasmosis and fetal research:	P 44:	
45. There is evidence that pretreatment of pregnant women influenced the resulto of the index test?	**1**. Yes **0.**No **-7.**No information	
46. Ratio diagnosis of acute maternal infection IgM:	P 46:	
47. Proportion of diagnosis of acute maternal infection avidity test:	P 47:	
48. Proportion of diagnosis of acute infection by maternal fetal U.S.:	P 48:	
49. Proporção de diagnóstico da infecção aguda materna por PCR:	P 49:	
50. Proportion of diagnosis of acute maternal infection clinic:	P 50:	
51. Study of HIV-infected pregnant women:	**1.** Yes **0.**No **-7.**No information	P 51:
52. Proportion of HIV-infected pregnant women in the sample:	P 52:	

Section 5: Test data			
53. Testing in-house?		**1.** Yes **0.**No **-7.**No information	P 53:
54. Name of the comercial test:			
55. Manufacturer of comercial test:		**1.** Yes **0.**No **-7.**No information	P 55:
56. Qualitative / Quantitative PCR		**1.** Qualitative **2.** Quantitative	P 56:
57. Name of the probe or primer:			P 57:
58. Amplified region:			P 58:
59. Reported detection limit and analytical sensitivity:			
60. Cutoff used in the test, if quantitative:			
61. Biological sample used:		**1.**Amniotic fluid **2.** Cord Blood **3.** Maternal Blood **4.** Other	P 61:
62a. Test performance in the first quarter:			P 62a:
62b. Test performance in the second quarter:			P 62b:
62c. Test performance in the third quarter::			P 62c:
63. This paper proposes a prediction model?			Yes **0.**No **-7.**No information
64. Test used as reference:		**1.** Follow **2.** Inoculation **3.** Placenta **4.**Outher	P 64:
65: Sample size:			
66a. True positive:	66b. False Negative:	66c. False positive:	66d. True negative:

Notes:
